# [^18^F]NOS PET Brain Imaging Suggests Elevated Neuroinflammation in Idiopathic Parkinson’s Disease

**DOI:** 10.3390/cells11193081

**Published:** 2022-09-30

**Authors:** Robert K. Doot, Anthony J. Young, Ilya M. Nasrallah, Reagan R. Wetherill, Andrew Siderowf, Robert H. Mach, Jacob G. Dubroff

**Affiliations:** 1 Division of Nuclear Medicine Imaging and Therapy, Department of Radiology in the Perelman, School of Medicine, University of Pennsylvania, Philadelphia, PA 19104, USA; 2 Department of Psychiatry, Perelman School of Medicine, University of Pennsylvania, Philadelphia, PA 19104, USA; 3 Department of Neurology, Perelman School of Medicine, University of Pennsylvania, Philadelphia, PA 19104, USA

**Keywords:** Parkinson’s, positron emission tomography, iNOS, microglia, neuroinflammation

## Abstract

Neuroinflammation is implicated as a key pathologic mechanism in many neurodegenerative diseases and is thought to be mediated in large part by microglia, native phagocytic immune cells of the CNS. Abnormal aggregation of the protein α-synuclein after phagocytosis by microglia is one possible neuropathophysiological mechanism driving Parkinson’s disease (PD). We conducted a human pilot study to evaluate the feasibility of targeting the inducible isoform of nitric oxide synthase using the [^18^F]NOS radiotracer to measure neuroinflammation in idiopathic PD. Ten adults consisting of 6 PD patients and 4 healthy controls (HC) underwent one hour of dynamic [^18^F]NOS positron emission tomography (PET) brain imaging with arterial blood sampling. We observed increased [^18^F]NOS whole brain distribution volume (V_T_) in PD patients compared to age-matched healthy controls (*p* < 0.008) via a 1-tissue compartment (TC) model. The rate constant K1 for transport from blood into tissue did not differ between groups (*p* = 0.72). These findings suggest elevated oxidative stress, a surrogate marker of inflammation, is present in early-stage idiopathic PD and indicate that [^18^F]NOS PET imaging is a promising, non-invasive method to measure neuroinflammation.

## 1. Introduction

The central nervous system’s immune response to disease, neuroinflammation, is modulated by microglia and has been implicated in a spectrum of neuropsychiatric diseases, including Alzheimer’s dementia, multiple sclerosis, addiction and major depressive disorder [1,2,3,4]. Parkinson’s disease (PD) is a progressive neurodegenerative disorder affecting over 2% of the population greater than 60 years old and is neuropathologically associated with aggregation of α-synuclein in Lewy bodies [5,6]. Inflammation has long been recognized as a component of PD pathology [7]. Many studies have shown that α-synuclein aggregates induce microglial activation [8,9,10]. In a prolonged inflammatory state, internalization of α-synuclein by activated microglia leads to oxidative stress and upregulation of iNOS, the inducible isoform of nitric oxide synthase [11,12,13]. Prior studies examining cerebral spinal fluid of PD patients, however, did not observe evidence of increased iNOS expression [14,15]. Yet, elevated iNOS has been implicated in PD pathogenesis) [16,17]. Prior postmortem histology in the midbrain, including the substantia nigra, has demonstrated elevated iNOS expression levels in individuals with idiopathic PD [18,19].

With growing evidence of an immunogenic component to the pathogenesis of PD, positron emission tomography (PET) brain imaging has been used to measure possible neuroinflammation in PD [20,21]. Most of these human PET studies focused on neuroinflammation have used radiotracers targeting the translocator protein (TSPO), also known as the peripheral benzodiazepine receptor [22]. While some studies have reported increased TSPO binding and inferred an increase in neuroinflammation, others have not [23,24]. Heterogeneity of these results could be attributed to limitations of the TSPO ligands, most of which have significant dependence of binding levels on a single nucleotide polymorphism (SNP), which thereby necessitates a complicated PET quantitation method [25,26]. Microglia activation is also not unique to inflammation; therefore, TSPO PET radiotracer uptake may not be entirely specific for inflammation [27,28].

PET imaging has been developed to measure other molecular targets implicated in inflammation, including the inducible isoform of nitric oxide synthase (iNOS) [27,29,30]. In its first human application, elevated [^18^F]6-(1/2)(2-fluoropropyl)-4-methylpyridin-2-amine ([^18^F]NOS) myocardial uptake predicted cardiac transplant rejection and correlated with iNOS immunohistochemistry [31]. Huang and colleagues bronchoscopically instilled endotoxin unilaterally into the lungs of healthy volunteers and found elevated PET distribution volumes, which correlated with bronchoalveolar lavaged iNOS immunohistochemistry [32]. We performed a pilot study to determine the feasibility of using [^18^F]NOS PET brain imaging to measure neuroinflammation in the setting of PD.

## 2. Materials and Methods

*Inclusion/Exclusion Criteria*: Six patients with a diagnosis of idiopathic Parkinson’s disease per UK brain bank criteria [33] as determined by a movement disorder neurologist (A.S.) were recruited and scanned with blood sampling under University of Pennsylvania Institutional Review Board-approved protocol NCT04062526 at the Hospital of the University of Pennsylvania in Philadelphia. All patients had a PD diagnosis for at least 3 years, were Hoehn and Yahr stage 2, and were free of dementia. Four healthy control subjects (HCs) without a history of epilepsy, head trauma, nicotine dependence, alcohol use disorder, or other neuropsychiatric disease and did not report use of a drug with CNS activity were also study participants and were pooled from three concomitant protocols (NCT04062526, NCT04274998, and NCT04401917). Subjects were scanned between October 2019 and August 2021. All subjects underwent identical [^18^F]NOS PET imaging procedures after providing written informed consent.

*Imaging Procedures*: Synthesis of [^18^F]NOS was performed as previously described [31,32]. Subjects were administered 165–228 MBq of [^18^F]NOS as an intravenous bolus and scanned for 60 min on an Ingenuity TOF PET/CT scanner (Philips Healthcare; Cleveland, OH, USA). Images were reconstructed using time of flight-ordered subset expectation maximization (TOF-OSEM) as described in Kolthammer et al. 2014 [34], into image frames as follows: 24 5-s frames, 6 × 10 s, 3 × 20 s, 2 × 30 s, 5 × 60 s, 10 × 300 s. During a separate visit, subjects underwent MRI brain imaging to obtain an MPRAGE T1 sequence with isotropic 1 mm voxels for co-registration and segmentation of the PET brain data.

*Arterial Blood Sampling and Analysis:* Arterial sampling was performed approximately every 15 s for the first 2 min and then at approximately 3, 4, 5, 10, 15, 30, and 60 min post-injection. Activity concentrations in whole blood and plasma were counted using a WIZARD2 2480 gamma counter (Perkin Elmer; Waltham, MA, USA). Acetonitrile-treated plasma supernatant was analyzed in a 1260 Infinity Series (Agilent Technologies; Santa Clara, CA, USA) high-performance liquid chromatology system using an Agilent ZORBAX StableBond C18 column via a mobile phase of 73% 0.1 M ammonium formate buffer and 27% methanol.

*Image Analysis:* PET images were corrected for inter-frame motion in PMOD version 3.7 (PMOD Technologies Ltd., Zurich, Switzerland). MR brain segmentations were processed using MRICloud and imported into Integrated Data Analysis Environment (IDAE) software for co-registration of PET and MR images and generation of time activity curves (TACs) for the whole brain and amygdala, caudate nucleus, cerebellum, cingulate, frontal lobe, hippocampus, insula, medulla oblongata, midbrain, occipital lobe, parietal lobe, pons, putamen, temporal lobe, temporal pole, thalamus, ventral striatum, entorhinal area, and parahippocampus regions [35,36]. Area under time activity curves (AUC) were measured using GraphPad Prism 8 (GraphPad Software, San Diego, CA, USA).

Kinetic analysis of [^18^F]NOS uptake was performed using PMOD version 3.7. Whole brain and regional TACs were fitted using each individual’s arterial blood activity, plasma partitioning, and sigmoid-fitted plasma parent fraction corrected for plasma protein binding. One-tissue compartment (1TC) and Logan Plot models of reversible radiotracer uptake with blood inputs were fitted with fixed blood volumes of 0.05 to estimate total volume of distribution (V_T_) for both models plus transport of radiotracer from blood into brain tissue (K1) for the 1TC model [37]. Blood delay terms were fitted for the 1TC model. The free fraction of [^18^F]NOS in plasma, f_p_, was not measured and assumed to be equal to PMOD’s kinetic modeling module’s default value of 1 as fp “is experimentally difficult to measure” accurately [38]. Since this study focused on examining differences between Vt measures of [^18^F]NOS uptake by PD and healthy control subjects, setting fp =1 avoided the addition of individual measurement error incurred by individual fp values, which have no impact on fitting model curves.

For fitting Logan Plots, we used a time t* of 20 min post-injection, when the tissue-blood relationship was linear within a 10% error for all participants. V_T_ parameter maps were created using PMOD version 3.7′s implementation of Alpert’s voxelwise 1TC model to show regional variability in uptake [39].

*Histology analysis:* Sections from a PD patient and an age-match control frontal cortex were fixed with 4% paraformaldehyde in phosphate-buffered saline (PBS) for 15 min and permeablized with 0.5% Triton X-100 in PBS for 20 min. The sections were blocked with 1.5% horse normal serum in PBS for 1 h and incubated with rabbit polyclonal Ab against human iNOS overnight at 4 °C. After washing, the sections will be incubated with FITC-conjugated anti-rabbit IgG secondary antibody for 1 h. The sections were coverslipped and imaged under a Zeiss AxioObserver Z1 microscope (Carl Zeiss Microscopy, LLC, Thornwood, NY, USA).

*Statistics:* Two sample two-tailed *t*-tests were used for comparisons of V_T_, K1, and AUC results with differences considered significant when *p* < 0.05 via GraphPad Prism 8 (GraphPad Software, San Diego, CA, USA). Spearman’s rank correlations were used for comparisons of PD patients’ Unified Parkinson’s Disease Rating Scale (UPDRS) scores with PET measurements.

## 3. Results

*Study Recruitment*: Ten subjects, six with PD (64 ± 4 yo) and four age-matched healthy controls (68 ± 13 yo), were enrolled and completed dynamic [^18^F]NOS PET scans with arterial blood sampling. Subject demographics and relevant markers for PD are reported in Table 1.

**Figure 1:**
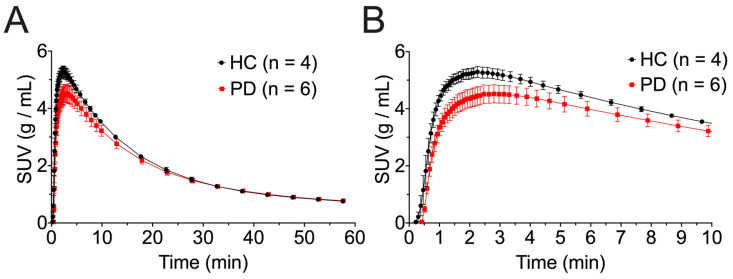
Whole-brain [^18^F]NOS time activity curves for healthy controls (HC) and PD patients from (**A**) 0–10 min post-injection and (**B**) 0–60 min post-injection. Curves are plotted as mean ± standard deviation.

*Brain Uptake of [^18^F]NOS*: Uptake of [^18^F]NOS typically peaked between 2 and 3 min post-injection, with washout over the remainder of the one-hour dynamic scan. Higher initial whole brain uptake was observed in HCs (Figure 1A), while PD patients exhibited a slower rate of washout than HC subjects, with brain uptake converging between the cohorts around 20 min post-injection (Figure 1B). No brain region was identified as being a suitable reference region free of specific [^18^F]NOS binding.

**Figure 2:**
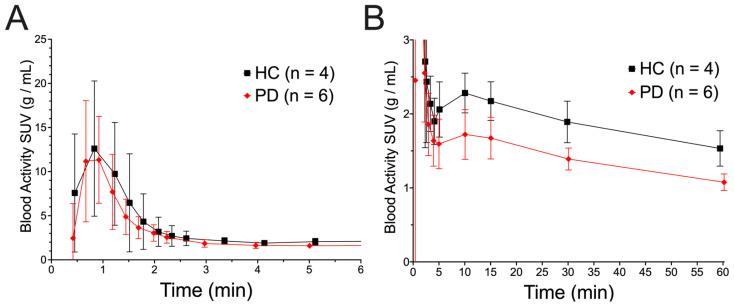
Arterial blood activity from (**A**) 0–6 min post-injection and (**B**) 0–60 min post-injection. Curves are plotted as mean ± standard deviation.

**Figure 3:**
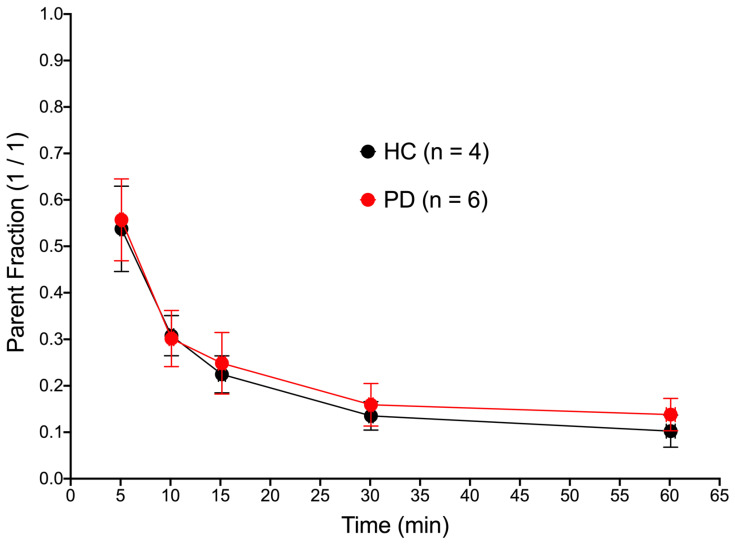
[^18^F]NOS parent fractions in plasma, corrected for plasma protein binding, plotted as mean ± standard deviation.

*Blood Activity Concentrations of [^18^F]NOS and Metabolites*: All ten subjects had full arterial blood sampling. Blood activity concentrations (Figure 2) were significantly lower in PD patients than HCs (PD AUC: 93.5 ± 4.5 g/mL·min, HC AUC: 122.4 ± 7.1 g/mL·min, *p* < 0.0001). This difference was observed most notably in the late blood samples (Figure 2B). [^18^F]NOS was rapidly metabolized with about 30% parent radiotracer remaining in arterial plasma around 10 min post-injection (Figure 3), with metabolism occurring at similar rates in both HC and PD cohorts.

**Figure 4:**
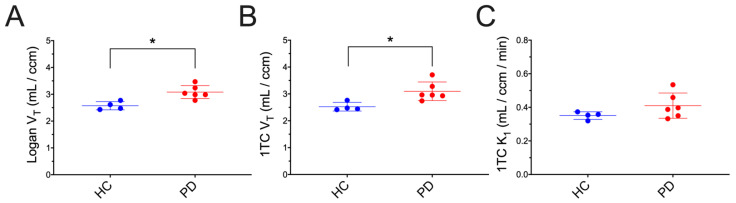
Whole-brain [^18^F]NOS Logan V_T_ (**A**), 1TC V_T_ (**B**) and 1TC K_1_ (**C**) comparisons between healthy and PD cohorts where an * denotes significant differences at *p* < 0.05.

**Figure 5:**
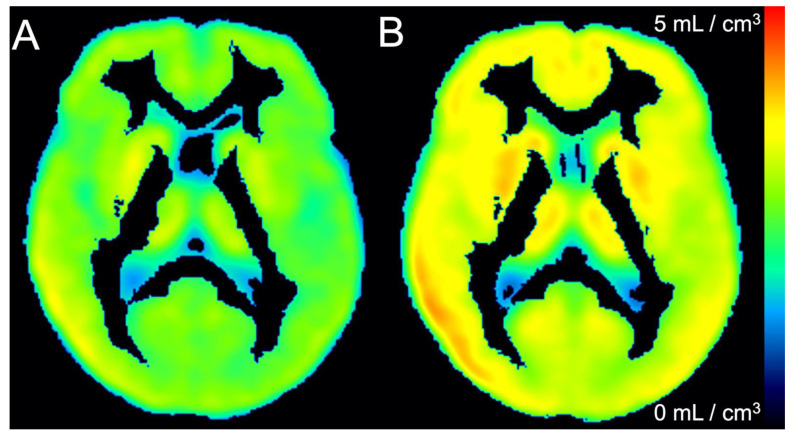
Mean one tissue compartment V_T_ parameter maps of (**A**) healthy controls and (**B**) Parkinson’s disease patients.

*Kinetic Analysis*: Whole brain [^18^F]NOS activity was well fit by models of reversible uptake, including 1TC r^2^ = 0.97 ± 0.02 and the Logan Plot r^2^ = 0.9999 ± 0.0001). V_T_ calculated by 1TC and Logan Plot models were highly correlated (r^2^ = 0.97). Both graphical and 1TC models found significant whole-brain differences between HC and PD cohorts, including t-test comparisons of whole-brain Logan V_T_ (*p* = 0.004) and 1TC V_T_ (*p* = 0.008), as shown in Figure 4. V_T_ differences between HC and PD cohorts were also observed in all 19 brain regions (*p* < 0.01). No significant whole brain difference was observed in K1 rate constants of [^18^F]NOS transport from blood to tissue (*p* = 0.72). V_T_ parameter maps for each cohort showing regional variability are depicted in Figure 5. Spearman’s rank correlation coefficient did not reveal a significant relationship between UPDRS scores, including subscores and total score, and 1TC whole brain [^18^F]NOS V_T_ (Supplementary Material).

**Figure 6:**
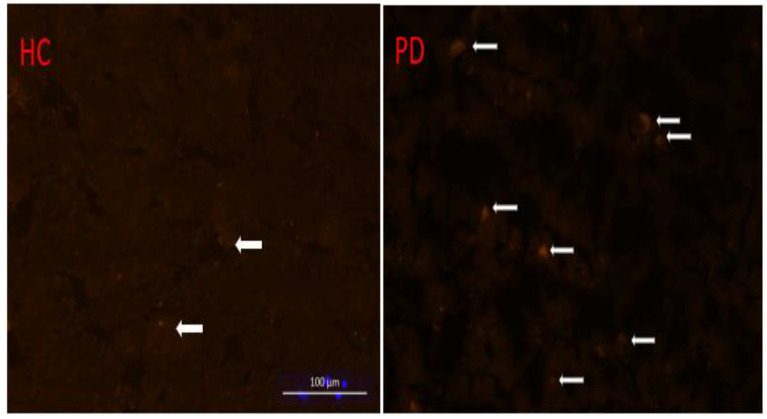
iNOS histology of the middle frontal gyrus showing greater expression in PD (**right**) compared to a healthy control (**left**). White arrows indicate regions of iNOS expression. The authors wish to acknowledge the late Dr. John Trojanowski for this figure.

*Histology studies.* Histology studies conducted on a PD and age-matched control frontal cortex are shown in Figure 6.

## 4. Discussion

This study is the first investigation of neuroinflammation using the PET imaging agent [^18^F]NOS [29,30]. [^18^F]NOS has previously been used to measure acute inflammatory responses in hearts of transplant patients and healthy subjects with endotoxin lung injury [31,32]. In a small cohort of 6 PD patients and 4 age-matched HCs, our findings of increased [^18^F]NOS V_T_ in the brain for PD patients suggest that [^18^F]NOS PET may provide a biomarker for oxidative stress in chronic neurologic disease and has potential for in vivo quantification and monitoring of inflammatory disease progression.

Observations of significant differences in V_T_ but no significant differences in K1 between cohorts indicate the greater [^18^F]NOS binding in PD patients than in HCs is not due to differences in transport of radiotracer between the cohorts. Our data also show depressed [^18^F]NOS activity in PD patient plasma. One possible explanation for this finding is extra-CNS inflammatory pathophysiology in PD patients. Previously, animal models of neurological disease using intraperitoneal injections elicit robust neuroimmunogenic responses, such as LPS and kainic acid, have demonstrated diminished TSPO radiotracer parent fraction levels [23,24].

While there are promising signs that [^18^F]NOS PET can discern differences in oxidative stress in vivo, there are limitations to its use in this study. First, this study examines a limited number of individuals (6 PDs and 4 HCs). It is important to note that no matter the modeling approach to determine [^18^F]NOS V_T_ (1-tissue compartment (TC) model or the more robust Logan graphical technique), the variability of the HC V_T_ measurement remains relatively small (standard deviations of 0.16 mL/ccm and 0.15 mL/ccm for 1TC and Logan, respectively) compared to that of PD (0.35 mL/ccm and 0.24 mL/ccm, respectively). For 1TC K1, the standard deviations were 0.023 mL/ccm/min for HC versus 0.075 for PD patients. Tight distributions of metrics in control populations offer unique opportunities in future study design. These results merit expansion to a larger cohort for each experimental group (*n* = 10) to address potential statistical power issues and offer an opportunity to examine additional questions raised by these data.

Data examining in vivo histological iNOS expression will also be of great value in both supporting this relationship and examining possible regional expression differences to both correlate with imaging findings and corroborate prior studies, though they focused largely on the substantia nigra [18,19]. Prior [^18^F]NOS PET imaging studies examining cardiac and pulmonary inflammation did demonstrate a strong histological correlation [31,32]. As suggested by alpha synuclein accumulation in Lewy bodies and behavorial changes, specific neuronal pathways have been implicated in PD pathogenesis [40]. While the 19 individual volumes of interest we included mirror the whole-brain results, our PET instrumentation’s resolution has intrinsic limits that could be informed by pathology [34]. Furthermore, the relationship between disease severity and neuroinflammation requires future attention. In our limited sample of idiopathic PD patients with the same Hoehn and Yahr stage (2), Spearman’s rank correlation coefficient did not demonstrate a significant relationship between UPDRS scores and 1TC whole brain [^18^F]NOS V_T_ (Supplementary Material). Examining PD patients at different Hoehn and Yahr stage as well as longitudinal studies of individual patients could provide insights into disease natural history. Additionally, such studies could help determine if neuroinflammation drives neurodegeneration or is simply a by-product of Parkinson’s disease.

Since no suitable brain reference region was identified in this study, future PD studies will at least initially require dynamic PET acquisition with blood sampling. Quantifying [^18^F]NOS uptake outside the brain may also likely benefit from blood sampling and metabolite analysis, as preliminary results in lung inflammation (Wetherill et al.) found promising differences in non-displaceable binding potential using a 2-tissue compartmental model in the lung [41].

Future work will include determining if image-derived input functions combined with venous blood samples can be used in place of more invasive arterial blood samples to estimate regional uptake of [^18^F]NOS.

## 5. Conclusions

[^18^F]NOS represents a promising imaging agent for microglial-mediated iNOS neuroinflammation pathways implicated in Parkinson’s Disease. Compared to age-matched healthy controls, PD patients had increased retention of [^18^F]NOS in the brain, suggesting higher oxidative stress and elevated levels of neuroinflammation. Additional investigations of [^18^F]NOS and other inflammation markers, including whole-body dynamic [^18^F]NOS PET/CT, may yield greater context for these findings and enable examination of wider applications of [^18^F]NOS as a biomarker for inflammation.

## Figures and Tables

**Table 1:** Subjects’ characteristics.

Patient ID *	M/F ^†^	Age	Ht ^‡^	Wt ^§^	IA ^‖^	UPDRS ^¶^	UPDRS ^¶^	UPDRS ^¶^	UPDRS ^¶^	UPDRS ^¶^	Anti-Parkinson’s
		(y)	(cm)	(kg)	(MBq)	NM-EDL ^#^	M-EDL **	ME ^∆^	MC ^√^	Total	Medications
Parkinson’s Disease Patients Cohort											
833589-02	F	65	150	52	224	1	0	15	2	18	carbidopa/levodopa
											rasagaline
833589-05	F	54	165	52	184	18	11	26	4	59	carbidopa/levodopa
											selegiline
833589-06	M	67	179	93	228	12	2	27	4	45	carbidopa/levodopa
											amantadine
833589-07	M	63	170	74	218	5	4	25	3	37	carbidopa/levodopa
											selegiline
833589-09	M	63	193	111	193	9	1	20	9	39	carbidopa/levodopa
											rasagaline, rotigotine
833589-11	M	70	186	82	165	2	8	33	0	43	carbidopa/levodopa
											rasagaline
Healthy controls cohort											
842717-6D	M	54	179	73	223	N/A	N/A	N/A	N/A	N/A	N/A
834090-04	M	74	177	127	227	N/A	N/A	N/A	N/A	N/A	N/A
833589-10	F	62	157	84	217	N/A	N/A	N/A	N/A	N/A	N/A
834090-01	M	83	170	73	213	N/A	N/A	N/A	N/A	N/A	N/A

* Identification number for consented patients who successfully completed [^18^F]NOS imaging session. ^†^ Male or female gender. ^‡^ Height (cm). ^§^ Weight (kg). ^‖^ Injected [^18^F]NOS activity (MBq). ^¶^ Unified Parkinson’s Disease Rating Scale (UPDRS). ^#^ Non-Motor aspects of Experiences of Daily Living (NM-EDL). ** Motor aspects of Experiences of Daily Living (M-EDL). ^∆^ ME Motor Examination. ^√^ MC Motor Complications.

## Data Availability

Not applicable.

## Supplementary Materials

https://www.mdpi.com/article/10.3390/cells11193081/s1. Supplementary Table S1: Spearman’s rank correlation of UPDRS scores with 1TC whole brain [^18^F]NOS V_T_ did not reveal significance between radiotracer binding (V_T_) with Parkinson’s disease severity measurements, total or subscores.
