# Supplementary material for: [18F]NOS PET Brain Imaging Suggests Elevated Neuroinflammation in Idiopathic Parkinson’s Disease
Source: Cells. 2022 Sep 30;11(19):3081. doi: 10.3390/cells11193081 (PMC9563966; doi:10.3390/cells11193081)
Supplement: Supplementary file 1 [file cells-11-03081-s001.zip › cells-1921026-supplementary.pdf]

Supplementary Materials:

UPDRS vs 1TC WB VT	Spearman's rho	*p*
Non-motor EDL	0.54	0.3
Motor EDL	-0.09	0.92
Motor Examination	0.09	0.92
Motor Complications	0.41	0.43
Total Score	0.54	0.3

Supplemental Table S1: Spearman's rank correlation of UPDRS scores with 1TC whole brain [^{18}F]NOS V_T did not reveal significance between radiotracer binding (V_T) with Parkinson's disease severity measurements, total or subscores.
